# Preventive Effect of Depolymerized Sulfated Galactans from *Eucheuma serra* on Enterotoxigenic *Escherichia coli*-Caused Diarrhea via Modulating Intestinal Flora in Mice

**DOI:** 10.3390/md19020080

**Published:** 2021-02-01

**Authors:** Yu Ma, Qian Zhang, Wenqiang Liu, Zhaohua Chen, Chao Zou, Linglin Fu, Yanbo Wang, Yixiang Liu

**Affiliations:** 1 College of Food and Biological Engineering, Jimei University, Xiamen 361021, China; my358108@163.com (Y.M.); zhangqian@jmu.edu.cn (Q.Z.); lwq19950512@163.com (W.L.); zhchen@jmu.edu.cn (Z.C.); zouchaoknight@163.com (C.Z.); wangyb@mail.zjgsu.edu.cn (Y.W.); 2 School of Food Science and Biotechnology, Zhejiang Gongshang University, Hangzhou 310018, China; fulinglin@zjgsu.edu.cn

**Keywords:** *Eucheuma serra*, sulfated polysaccharide, enterotoxigenic *Escherichia coli*, bacterial diarrhea, 16S rRNA, gut microbiota

## Abstract

In this work, the preventive effect of depolymerized sulfated polysaccharides from *Eucheuma serra* (DESP) on bacterial diarrhea by regulating intestinal flora was investigated in vivo. Based on the enterotoxigenic *Escherichia coli* (ETEC)-infected mouse diarrhea model, DESP at doses ranging from 50 mg/kg to 200 mg/kg alleviated weight loss and decreased the diarrhea rate and diarrhea index. Serological tests showed that the levels of inflammation-related factors were effectively suppressed. Furthermore, the repaired intestinal mucosa was verified by morphology and pathological tissue section observations. Compared with the model group, the richness and diversity of the intestinal flora in the DESP group increased according to the 16S rRNA high-throughput sequencing of the gut microbiota. Specifically, *Firmicutes* and *Actinobacteria* increased, and *Proteobacteria* decreased after DESP administration. At the family level, DESP effectively improved the abundance of *Lactobacillaceae*, *Bifidobacteriaceae*, and *Lachnospiraceae*, while significantly inhibiting the growth of *Enterobacteriaceae*. Therefore, the antimicrobial diarrhea function of DESP may be related to the regulation of intestinal microbiota.

## 1. Introduction

As one of the most common diseases with a global incidence of more than 1 billion per year, diarrhea can cause 600,000 deaths in most underdeveloped countries and regions each year, mainly children under five years of age [1,2]. Enterotoxigenic *Escherichia coli* (ETEC) is considered the most common cause of diarrhea [3]. It is believed that ETEC can secrete specific adhesion proteins or peptides, allowing it to firmly adhere to and colonize small intestinal epithelial cells (IECs) [4]. ETEC also produces a large amount of enterotoxin, mainly heat-stable enterotoxins (ST) and heat-labile toxin (LT), that alters the integrity of tight junctions in host small IECs, destroys the internal environment of fluids, and stimulates excessive secretion of body fluids and electrolytes [5]. Currently, oral rehydration salt and antibiotics represent the primary treatment methods for infectious diarrhea. As the first-line treatment for diarrhea worldwide, oral rehydration therapy is safe and convenient but cannot reduce the duration and severity of the condition [6]. Antibiotics have been considered one of the most effective treatments in the past, but long-term use results in drug resistance and intestinal flora imbalance and can even cause the recurrence of diarrhea [7]. Therefore, increasing attention has been focused on natural active substances.

Sulfated polysaccharides, the main active ingredient in seaweed, have been confirmed to exhibit a variety of antiviral [8], anti-inflammatory [9], anticoagulant [10], and anticancer [11] physiological activity. In recent years, the anti-diarrheal activity of sulfated polysaccharides has received widespread attention. For example, sulfated polysaccharides derived from *Gracilaria intermedia* and *Hypnea musciformis* exhibit an ameliorative effect on acute and secretory diarrhea induced by castor oil and cholera toxin, respectively, by increasing the Na^+^/K^+^-ATPase activity in the small intestine and reducing gastrointestinal transit and intestinal fluid accumulation [12,13]. It is also believed that sulfated polysaccharides from red algae, *Porphyra haitanensis*, and *Gracilaria lemaneiformis* can inhibit proinflammatory release cytokines, IL-6 and TNF-α, as well as the secretion of immunoglobulin A (IgA), in ETEC-K88-induced mouse diarrhea [14]. In addition, studies have also shown that sulfated seaweed polysaccharides exhibit an anti-diarrhea effect by binding with monosialoganglioside-1 and cholera toxin, blocking their attachment to the enterocyte cell surface [15].

In fact, there is growing evidence that the anti-diarrhea effect involves the regulation of intestinal flora [16]. It was reported that probiotic supplementation in children with acute infectious diarrhea caused by rotavirus infection caused the diarrhea symptoms to cease, and the maladjusted intestinal flora even returned to a normal state [17]. The observation results regarding the intestinal flora changes in newborn calves over time revealed that *Ruminococcus 2*, *Trueperella*, *Dorea*, *Streptococcus*, and *Erysipelatoclostridium* could be used as key microbial markers to predict early diarrhea since the accuracy was as high as 84.3% [18]. During the treatment of antibiotic-related diarrhea in rats using herbal formula, the abundance of *Sutterella* was significantly suppressed, which was thought to be a pivotal phylotype in improving diarrhea [19]. Therefore, the intestinal flora is closely related to the occurrence and development of diarrhea. Improving the composition of gut microflora should be a crucial strategy in preventing or treating infectious diarrhea.

*Eucheuma serra* (*E. serra*) is traditional edible red algae from China’s southeast coastal area and is the primary raw material for producing carrageenan [20]. The sulfated galactan is a vital bioactive substance in *E. serra*, and approximately 90% of the linear backbone is composed of alternating 3-linked β-D-galactopyranos and 4-linked α-D-galactopyranos residues [21]. Previous research found that the depolymerized sulfated polysaccharide from *E. serra* (DESP) inhibited the growth and adhesion of ETEC K88, but not probiotics [22], while it may be therapeutic to bacterial diarrhea. Therefore, this study aims to investigate the preventive effect of DESP on ETEC-K88-induced diarrhea. Furthermore, the structural shifts of the gut microbiota in response to DESP treatment are also discussed. This research may provide information for the future development of anti-diarrheal functional foods and feeds.

## 2. Results

### 2.1. The Effect of DESP on the Body Weight and Diarrheal Symptoms

**Figure 1:**
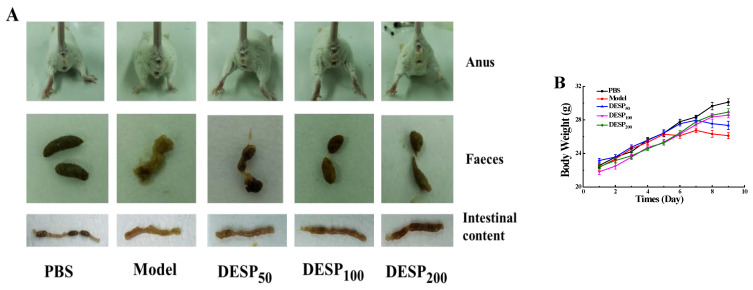

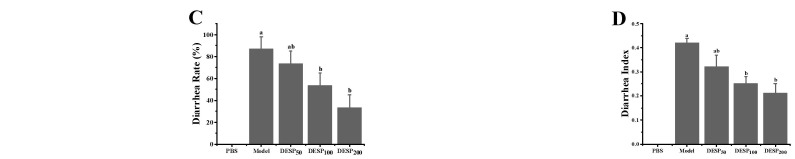
The improving effect of *Eucheuma serra* (DESP) on diarrhea symptoms in mice. (**A**). Anal, fecal, and intestinal content observations (**B**). Body weight changes during this trial (**C**). The effect of DESP administration on the diarrhea rate (**D**). a,b: bars with different letters represent significant differences. The effect of DESP administration on the diarrhea index (*p* < 0.05).

As shown in Figure 1A–D, the clinical symptoms and anatomical observation of the diarrhea mice were detected before and after DESP administration. Compared with the model group, the mice in the PBS group displayed good mental state, strong mobility, smooth and shiny fur, as well as stable weight gain. Furthermore, lumpy intestinal contents, a clean anus, granular feces, and stable rectal temperature (around 37.5 °C) were also observed. However, the model group mice showed depression, decreased mobility, dull coats, reduced eating ability, and slightly decreased average weight. The diarrhea rate and diarrhea index of mice in the model group were 86.67% and 0.42, respectively. The feces in the model group were black-red, with light yellow purulent mucus, the jejunum was shriveled, and the contents were yellow and thin. The diarrhea rate and diarrhea index of mice with continuous intragastric administration of 200 mg/kg DESP were 33.34% and 0.21, respectively, which was only 38.47% and 50.00% in the model group, indicating that polysaccharides prevented bacterial diarrhea. 

### 2.2. Morphological and Histological Observation of Intestinal Inflammation

**Figure 2:**
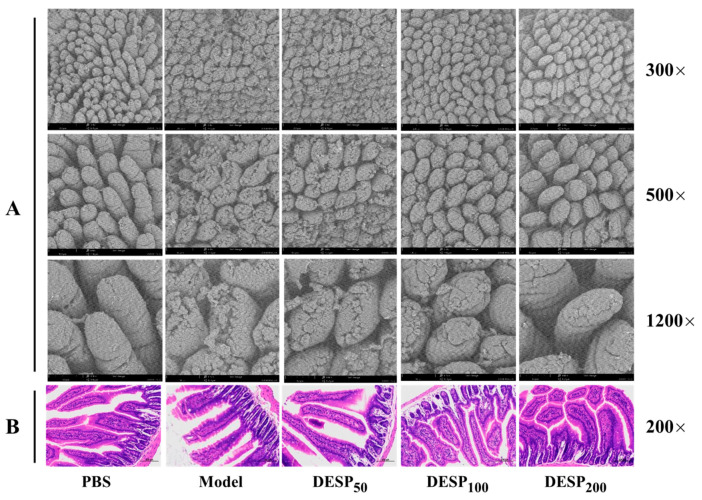
The effect of DESP administration on the intestinal inflammation in diarrhea mice based on morphological and histological observations. (**A**) SEM observation of the integrity of jejunum villi. (**B**) Optical microscope observation of the jejunum tissue after hematoxylin-eosin (HE) staining.

In this work, scanning electron microscopy (SEM) was used to observe the integrity of the jejunum villi and observe the pathological sections of the jejunum tissue. Based on the SEM observations, Figure 2A shows that the intestinal villi in the PBS group were arranged neatly, distributed evenly, and densely, and displayed a plump, round shape without cracks or defects. Different from the PBS group, the integrity of the jejunum villi in the model group was obviously destroyed, displaying a ruptured surface and increased damage. After DESP prevention treatment ranging from 50 mg/kg to 200 mg/kg, the jejunal villi were only slightly damaged, without breakage or shedding. Especially at 200 mg/kg, the morphology of the jejuna villi in the ETEC-K88 injured mice was similar to that of normal mice.

The pathological observation results of the jejunum tissue are shown in Figure 2B. In the PBS group, the epithelial cells were normal and closely arranged, and an intact intestinal mucosal structure was displayed. In addition, the columnar cells and goblet cells in the lamina propria were abundant and no apparent signs of inflammation were observed. Whereas, in the model group, the villi were atrophied and severely widened, and prominent inflammation was evident. When the dose of DESP was 50 mg/kg or 100 mg/kg, a slight widening of the spaces between the intestinal villi and mild inflammation was apparent. Nevertheless, when the DESP dose increased to 200 mg/kg, intestinal tissue cells appeared normal and were closely arranged, with no visible inflammation. The intestinal morphology of the mice closely resembled that of the normal mice in the PBS group. These results indicate that DESP is effective in inhibiting the damage to IECs caused by diarrhea.

### 2.3. Serological Analysis of the Diarrhea Symptoms in the Mice

**Figure 3:**
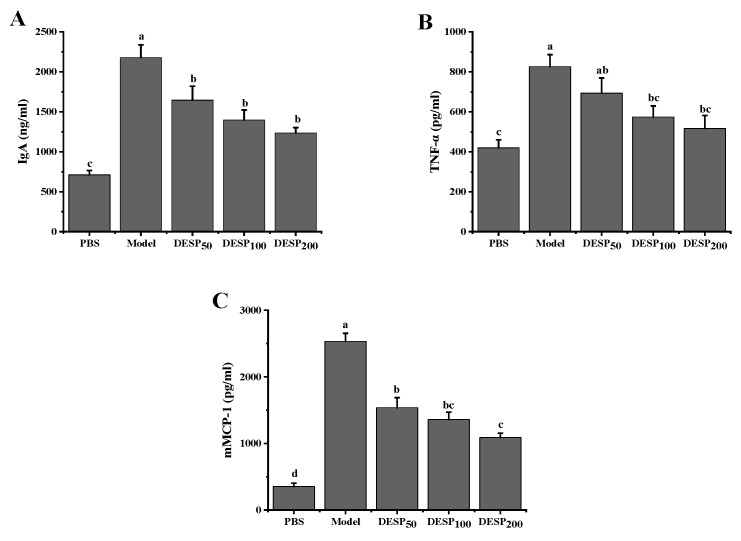
Serological analysis of diarrhea symptoms in mice before and after DESP treatments at doses of 50 mg/kg to 200 mg/kg. (**A**) IgA; (**B**) TNF-α; (**C**) mMCP-1; a–d: bars with different letters represent significant differences (*p* < 0.05).

According to the changes in serum levels of IgA and some inflammatory factors such as tumor necrosis factor-a (TNF-α) and mouse mast cell protease (mMCP)-1, the anti-inflammatory effects of DESP on mice were studied. ETEC-K88 stimulation resulted in a significant increase in the IgA antibody levels, as well as the TNF-α and mMCP-1 inflammatory factors in the mice’s serum (Figure 3). However, after the polysaccharide administration, the IgA, TNF-α, and mMCP-1 serum levels decreased significantly (*p* < 0.05) in a dose-dependent manner. After the mice were given 50 mg/kg DESP, the IgA, TNF-α, and mMCP-1 serum levels decreased by 24.41%, 16.02%, and 39.29%, respectively. However, when the DESP dose was increased to 200 mg/kg, the IgA, TNF-α, and mMCP-1 serum levels decreased by 43.26%, 37.42%, and 57.02%, respectively. These results further indicated that DESP prevented the inflammation in the diarrhea mice to some extent.

### 2.4. The Alteration of the Alpha Diversity of the Gut Microbiota by DESP 

**Figure 4:**
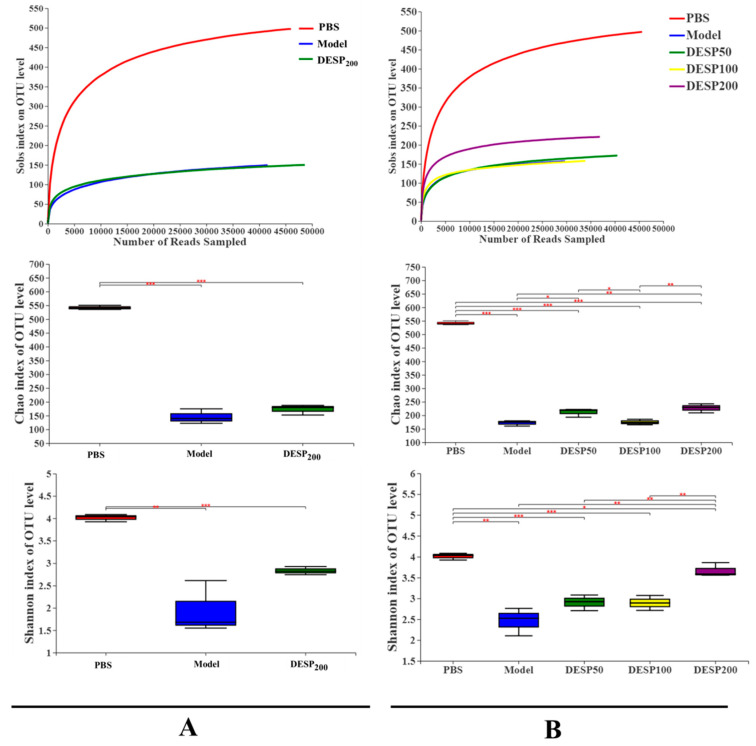
Changes in the diversity of the fecal microbial flora. (**A**). The OTU levels, Chao1 index, and Shannon index in the feces after consuming water containing SM for 36 h. (**B**). The OTU levels, Chao1 index, and Shannon index in the feces 1 h after the last bacterial donation. *, *p* < 0.05; **, *p* < 0.01, ***, *p* < 0.001.

Figure 4A shows that after 36 h of streptomycin (SM) interference, the operational taxonomic unit (OUT) level in the feces was significantly decreased, even in mice that received a DESP dose of 200 mg/kg. On the basis of the Chao1 estimate of the bacterial communities and the Shannon index assessment of species diversity, the abundance of the observed bacterial communities and diversity indexes in the model group was significantly lower than in the PBS group (*p* < 0.001 and *p* < 0.01). However, after 200 mg/kg DESP treatment, the species abundance and diversity index increased slightly compared with the model group, but no significant differences were evident. The above results indicate that DESP treatment can enhance the ability of the intestinal flora to resist interference from antibiotics.

After ETEC-K88-induced diarrhea, the OTU level, species richness, and the fecal diversity of the mice in each group were affected (Figure 4B). Specifically, compared with the PBS group, the OTU level in the model group declined by 69.86%, while the observed number of species and the Shannon index value showed a significant decrease (*p* < 0.001 and *p* < 0.01). Different from the model group, the OTU level, species richness, and fecal diversity of the mice in the polysaccharide prevention group showed an increasing trend. Specifically, the Shannon index in the 200 mg/kg group was close to the normal level, indicating that the high DESP treatment dose can effectively maintain the balance of the intestinal flora.

### 2.5. The effect of DESP on the Intestinal Flora Compositions

**Figure 5:**
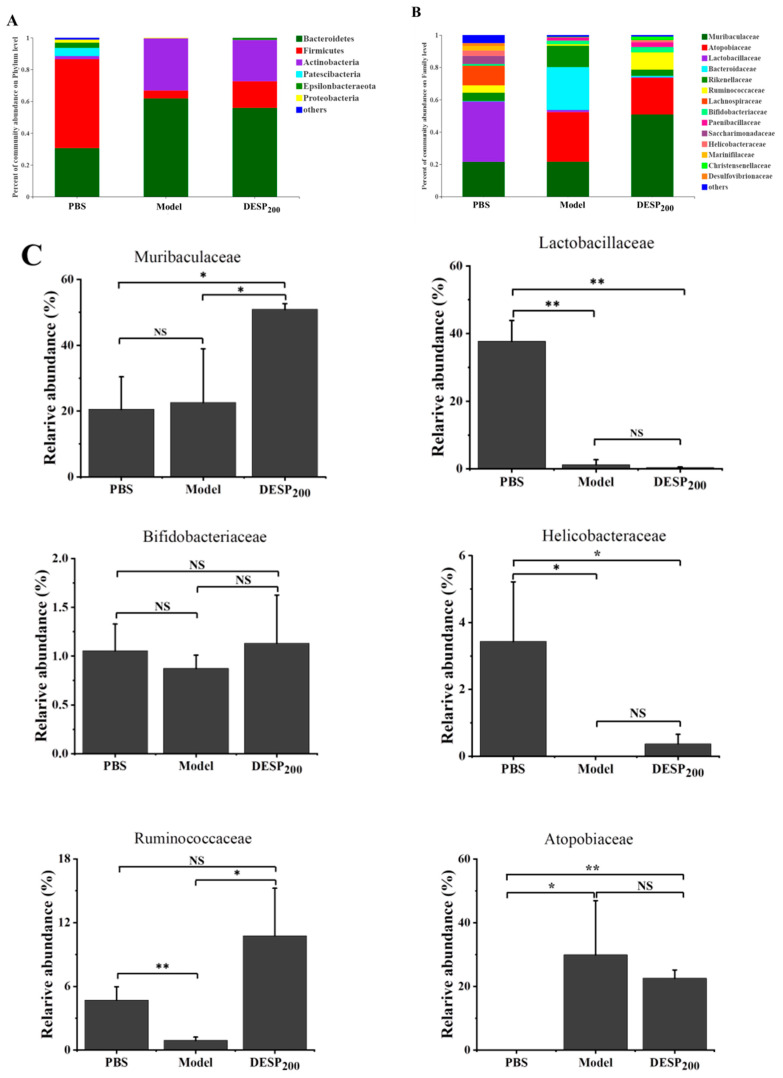
The composition of the mouse gut microbes after SM interference. (**A**) The abundance of the primary phylum level. (**B**) The abundance of the primary family level. (**C**) The bacterial content differed significantly among the groups. *, *p* < 0.05; **, *p* < 0.01; NS, no significance.

As the balance of the intestinal flora is disturbed by antibiotics, the intestinal flora state was determined after 36 h of antibiotic use. Remarkable shifts in the microbial composition in the gut were revealed at the phylum (Figure 5A) and family (Figure 5B) levels in response to SM treatment. The high-quality data were sufficient to represent all species in the community. In the PBS group, *Bacteroidetes* and *Firmicutes* were the dominant flora, accounting for 28.51% and 36.55%, respectively. After SM interference, the abundance of *Bacteroides* reached 61.87% and the *Actinobacteria* levels increased from 3.85% to 32.82%. Compared with the PBS group, the group exposed to 200 mg/kg DESP displayed higher *Bacteroides/Actinobacteria* content and reduced *Firmicutes* levels. Figure 5C shows that after SM treatment, the abundance of *Lactobacilleae* (*p* < 0.01), *Helicobacteraceae* (*p* < 0.01), and *Ruminococcaceae* (*p* < 0.05) decreased significantly in the model group, while the abundance of *Bifidobacteriaceae* was lower throughout with no statistical differences. The abundance of *Muribaculaceae* and *Ruminococcaceae* was higher in the DESP-treated mice than in the model group (*p* < 0.05). Notably, SM treatment specifically enhanced the relative abundance of *Atopobiaece* (*p* < 0.05).

**Figure 6:**
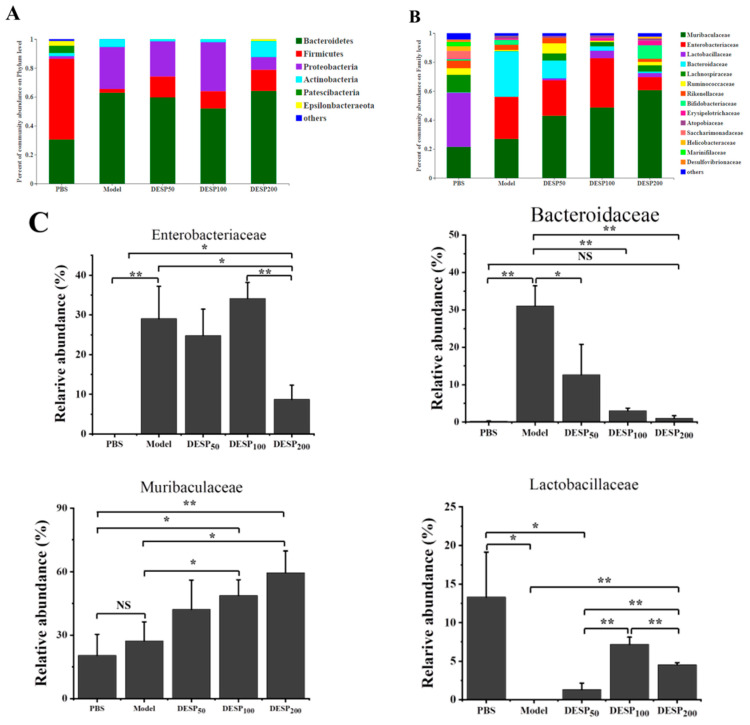

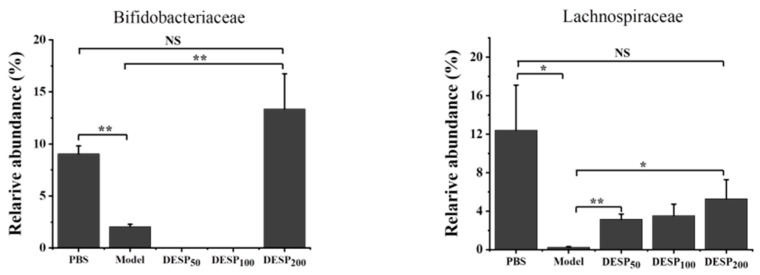
DESP treatment changes the composition of mouse intestinal flora. (**A**) The abundance of the primary phylum level. (**B**) The abundances of the primary family level. (**C**) The bacterial content differed significantly among the groups. *, *p* < 0.05; **, *p* < 0.01; NS, no significance.

Feces were collected on the 10th day, 1 h after the last ETEC-K88 donation, to determine the composition of the intestinal flora. The taxon-based analysis was used to identify the marked differences at the phylum and family levels among the mice from different groups (Figure 6A,B). The abundance of *Firmicutes* and *Proteobacteria* in the gut microbial community of the model group increased, while the proportion of *Bacteroides* was lower. When the mice were supplemented with 200 mg/kg DESP, the proportion of *Actinobacteria* was 25.99%, and the total proportion of *Firmicutes* and *Bacteroides* reached 78.78%, resembling almost normal levels. Figure 6B,C shows that the related abundance of *Enterobacteriaceae* in the model group increased significantly after ETEC-K88 administration, reaching 29.05%. However, in the group exposed to a 200 mg/kg dose of DESP, the proportion of *Enterobacteriaceae* declined to 8.72%. DESP treatment increased *Muribaculaceae* and decreased *Bacteroidaceae*. The proportion of *Lactobacilleae* (*p* < 0.05), *Bifidobacteriaceae* (*p* < 0.01), and *Lachnospiraceae* (*p* < 0.05) were lower in the model group than in the PBS group. However, *Lactobacilleae*, *Bifidobacteriaceae*, and *Lachnospiraceae* increased in the polysaccharide prevention group, especially at a dose of 200 mg/kg. Furthermore, the abundances of *Lactobacilleae* and *Bifidobacteriaceae* were significantly increased compared to the model group (*p* < 0.01), and no significant differences were evident from the normal group. These results indicate that polysaccharides can improve the stability of the intestinal flora to a certain extent and reduce the damage caused by antibiotics. 

## 3. Discussion

In China, in order to improve human health and treat various diseases, plants have been used as herbal supplements and medicines for at least 2000 years. [23]. As the main component of most medicinal plants, polysaccharides have been reported to be involved in a variety of significant bioactivities, such as displaying antitumor, antioxidant, antidiabetic, radioprotective, hypolipidemic, and immunomodulatory properties [24]. In recent years, active polysaccharides have attracted widespread attention due to their anti-diarrhea activity. *Astragalus polysaccharides*, at a dose of 200 mg/kg, can significantly inhibit the *Salmonella typhimurium*-induced expression of toll-liked receptor 4 (TLR4) and myeloid differentiation factor 88 (MyD88) in the jejunum while further inhibiting nuclear factor-κB (NF-κB) activation and reducing intestinal inflammation and the symptoms of diarrhea in mice [25]. *Rheum tanguticum* polysaccharides extracted from rhubarb exhibited a protective effect on 2,4,6-trinitrobenzenesulfonic acid (TNBS) and induced diarrhea, inflammation, and colon damage in rats [26]. In addition, sulfated polysaccharides derived from marine seaweed, such as *Porphyra haitanensis, Eucheuma cottonii*, and *Gracilaria lemaneiformis*, also display inhibitory effects on antibacterial diarrhea in vivo [12,14]. Research has indicated that DESP has a significant preventive impact on bacterial diarrhea induced by ETEC-K88 at doses ranging from 50 mg/kg to 200 mg/kg.

As the leading cause of diarrhea in humans and young animals [27], the enterotoxin produced by ETEC can stimulate the secretion of chloride from the apical region of enterocytes by up-regulating the levels of cAMP and cGMP, reducing sodium absorption, and causing dehydration diarrhea [28]. The production of inflammatory mediators and the release of reactive oxygen species (ROS) resulting from ETEC infection can cause intestinal damage and cell death [29]. Studies have shown that ETEC infection can also change the gene expression of tight junction proteins, leading to increased intestinal permeability and affecting the integrity of the intestinal barrier function [30]. In this work, both intestinal inflammatory damage and the increase in serum inflammation-related factors have been observed. ETEC infection caused an abnormal increase in serum IgA. Some studies have reported that elevated serum IgA levels may be induced by commensal bacteria (*Proteobacteria*), providing a constitutive humoral shield to resist systemic bacterial invasion [31]. However, there is also evidence that some diseases will be accompanied by abnormally elevated serum IgA [32,33]. The increase in intestinal permeability induced by TNF-α, mainly produced by monocyte-macrophages, is considered a significant cause of various intestinal inflammations [34]. Although the involvement of mMCP-1 in allergic reactions is well documented, some reports indicate that mMCP-1 is partly mediated by the intestinal inflammation associated with gastrointestinal helminths. Therefore, blocking the release of mMCP-1 may be an effective way to prevent the intestinal inflammation associated with gastrointestinal helminths [35]. The results of this study showed that the DESP treatment groups all exhibited an intestinal protective effect against ETEC infection. The groups exposed to 200 mg/kg DESP supplementation, in particular, exhibited no significant differences from the normal group. In fact, the anti-inflammatory effect of seaweed sulfated polysaccharides has been reported in previous studies [9,36]. Moreover, studies have shown that inhibiting IL-6, IL-1, and IL-8 proinflammatory factors produced by IECs can also have an anti-diarrhea effect [37]. 

In recent years, growing research evidences have shown that intestinal flora imbalance plays a vital role in bacterial diarrhea. When foreign bacteria invade the intestines, the original diversity and abundance of intestinal microbes are affected, disrupting intestinal homeostasis. The importance of symbiotic bacteria in maintaining gastrointestinal homeostasis cannot be overemphasized. Gut microbiota is essential to many aspects of human health, including host metabolism, system immunity, and neurobehavioral traits [38]. The intestinal flora and its short-chain fatty acid (SCFAs) metabolites play an essential role in many inflammatory and immune diseases, such as arthritis, asthma, inflammatory bowel disease, allergic diseases, and colon cancer [39]. According to recent studies, DESP can specifically inhibit the growth of ETEC-K88 but have no such effect on probiotic bacteria [40], exhibiting the potential for treating bacterial diarrhea induced by ETEC-K88. Therefore, it is worth exploring the mechanism of DESP involved in bacterial diarrhea from the perspective of intestinal flora.

As drugs that are frequently used in clinics, antibiotics are prescribed to prevent and treat bacterial diarrhea. However, antibiotic treatment may disturb the colonization resistance of gastrointestinal flora and even cause the condition’s recurrence. In particular, antibiotics such as aminopenicillins, cephalosporins, and clindamycin that act on anaerobes are most commonly associated with diarrhea [41]. In addition to frequent watery bowel movements, urgency, and abdominal cramping, and antibiotic-associated diarrhea (AAD) is correlated with altered intestinal microflora [42]. In this study, OTU-based analysis showed that SM treatment reduced the microbial load of mice, while the alpha diversity of mouse gut microbes also decreased. Moreover, the abundance of many intestinal microbes is also significantly reduced, which may be related to the spectral bactericidal properties of SM [43]. This intestinal disorder resulting from antibiotics is considered the leading cause of AAD [44], which is why the restoration of the intestinal flora destroyed by antibiotics has become a new strategy for treating AAD. For example, Chinese yam can alleviate AAD symptoms by repairing the ampicillin-induced intestinal microbiota disorder, increasing the abundance of the intestinal probiotic *Lactobacilli* and *Bifidobacteria*, and decreasing the abundance of potential pathogenic *Clostridium* perfringens and *Enterococcus* [45]. *Panax ginseng* polysaccharides and *Astragalus* polysaccharides altered the diversity and composition of the gut microbiota in mice with AAD, induced by lincomycin hydrochloride, restored the gut microbiota, and promoted the metabolic balance and mucosal recovery [45,46]. 

However, the impact of various microbes on the gut and the host are quite different. For instance, *Lactobacillaceae* and *Bifidobacteriaceae* are the important resource of probiotics. Probiotics exert their beneficial effects on the host via four main mechanisms, namely improvement of barrier function, interference with potential pathogens, the production of neurotransmitters, and immunomodulation [47]. Furthermore, *Lactobacillus* and *Bifidobacterium* can produce lactic and acetic acid by metabolizing carbohydrates. The production of these organic acids can lower pH in the intestinal cavity and have an antagonistic effect on other pathogenic bacteria [48]. They do not produce butyrate but can increase the level of this substance and other SCFAs in the intestine by cross-feeding other symbiotic microbial flora [49]. SCFAs are an essential fuel for IECs and strengthen the gut barrier function [50]. *Lachnospiraceae* is the main butyrate-producing bacteria in SCFAs [51]. Butyrate has a significant anti-inflammatory impact, inducing the differentiation of regulatory T cells and reducing the levels of inflammatory factors and chemokine ligand 1, including inhibiting *Clostridium difficile* infection and the development of colitis [52,53]. In addition to *Lachnospiraceae*, the autochthonous and benign *Ruminococcaceae* species that primarily inhabit the caecum and colon is also a member of SCFA producers and responsible for the degradation of fibers and diverse polysaccharides [54,55]. Under normal circumstances, *Enterobacteriaceae* account for less than 1% in healthy intestines but increase significantly in inflamed intestines, which is a sign of intestinal flora imbalance [56]. Studies have shown that *Bacteroidaceae* is significantly enriched in infants with eczema and may participate in autoimmune diseases by promoting the secretion of IL-17 by Th17 cell [57]. Interestingly, the growth of these two Gram-negative bacteria was inhibited by the anionic polysaccharide DESP. Furthermore, previous research confirmed that DESP can specifically inhibit the growth of Gram-negative bacteria, ETEC, but has no noticeable effect on the growth of Gram-positive bacteria and yeast [40]. This study showed that, compared with the model group, DESP effectively enriched the abundance of *Lactobacillaceae*, *Bifidobacteriaceae*, and *Lachnospiraceae* and inhibited the growth of *Enterobacteriaceae* and *Bacteroidaceae* in intestinal, which is similar to the regulation of fucoidan on the intestinal flora [58]. This research indicated that DESP played a positive role in maintaining intestinal homeostasis. It further suggests that the anionic polysaccharide, DESP, may restrict the growth of Gram-negative bacteria and not be limited to ETEC. In addition, DESP also increases the abundance of the dominant bacterial group *Muribaculaceae*. This family is not only versatile with respect to complex carbohydrate degradation but is one of the stomach microbes involved in the expansion of type 2 innate lymphoid cells (ILC2s) that can produce secretory IgA (sIgA) to provide immune protection in the stomach [59,60]. In general, the modification effect of DESP on the intestinal flora is similar to other natural ingredients, including decreasing the number of harmful bacteria and increasing the relative abundance of *Firmicutes*, probiotics, and SCFA-producing bacteria, while improving the metabolic level and host immunity [45,46,61]. Furthermore, some natural active components, such as dietary polyphenols, can exhibit anti-diarrheal behavior by inactivating the LT produced by ETEC [62]. By contrast, probiotics are usually employed to treat bacterial diarrhea by promoting the expression of intestinal tight junction proteins and improving intestinal barrier function [30,63]. Although the anti-diarrheal effect of DESP and the improvement of intestinal flora are well understood, the specific mechanism of intestinal flora in improving bacterial diarrhea requires further exploration.

## 4. Materials and Methods 

### 4.1. Materials and Chemical Reagents 

The DEAE-Cellulose 52 from Sigma (Sydney, Australia) was used for separating depolymerized sulfated galactans. Imject Alum was provided by Thermo Fisher Scientific Inc (Waltham, MA, USA). The commercial enzyme-linked immunosorbent assay (ELISA) IgA kit was obtained from Abcam (Cambridge, UK). The ELISA kits of mouse mast cell protease (mMCP)-1 and TNF-αwere obtained from R&D Systems (Minneapolis, MN, USA). Other reagents were analytical reagent-grade and purchased from the China National Pharmaceutical Industry Corporation Ltd. (Shanghai, China). 

### 4.2. Purification and Preparation of DESP

The DESP was obtained according to the methods described in our previous studies [22,40]. Briefly, the sample was washed with tap water to remove sand, salt, and epiphytes. After being dried, the algae was ground into powder (40 mesh sieved) and stored at 4 °C. Subsequently, 100 g of the seaweed powder was extracted in a hot water at 55 °C for 4 h. Then, the *E. serra* syrup was filtered with a filter cloth. After being concentrated to 1/4th of the original volume, the cooled filtrate was precipitated overnight with three volumes of ethanol at 4 °C. Next, the collected precipitate was washed with 75% ethanol. After lyophilization, the crude *E. serra* sulfated polysaccharides was obtained. The Sevag method was employed to remove the proteins from the crude polysaccharides [64]. Subsequently, the ultrafiltration technology was used to remove the metal ions absorbed on the sulfated galactans. The crude polysaccharides were dissolved in distilled water and the final concentration was 0.5% (*w/v*). After the pH was adjusted to 5.0 by acetic acid, ethylenediaminetetraacetic acid (EDTA) disodium was added at a final concentration of 20.0 mmol/L. Then, the solution was stirred at 1200 r/min for 30 min and filtered using a 4 kDa ultrafiltration membrane. After eight ultrafiltration (dilution ratio was 8) cycles, the polysaccharide concentrate was collected for depolymerization.

The depolymerized sulfated galactan was prepared by high temperature and high pressure technology. The polysaccharide solution was hydrolyzed in an autoclave reactor (Shanghai Boxun Industry & Commerce Co. Ltd. Medical Equipment Factory, Shanghai, China) at 121 °C and 0.103 MPa for 40 min. After that, the depolymerized product was collected and fractionated by ultrafiltration. The fractions with a molecular weight (MW) ≤ 6kda were collected and lyophilized. 

### 4.3. Preparation of the Bacterial Suspension

The Gram-negative ETEC K88 (CN-3-321) was obtained from the Beijing Baiou Bowei Biotechnolgy Co., Ltd. (Beijing, China). The Luria–Bertani (LB) medium was used to cultivate the bacteria. After being incubated at 37 °C for 12 h, the bacterial suspension was centrifuged at 5000× *g* for 10 min. Subsequently, the pellets were washed twice and resuspended in sterile phosphate-buffered saline (PBS) to form about 10^9^ colony forming units (CFU) mL^−1^.

### 4.4. Animals and Experimental Design

All animal procedures were carried out following the Guidelines for Care and Use of Laboratory Animals by Jimei University (SCXK 2012–0005), and the animal experiments were approved by the Animal Ethics Committee of China. The ETEC-induced diarrhea model was established according to the method described earlier [63], with some modifications. The specific-pathogen-free (SPF) male ICR mice were five weeks old and 15 ± 2 g was obtained from the Shanghai Slac Laboratory Animal Co. Ltd (Shanghai, China). After a one-week acclimatization period, the mice were randomly divided into five groups (5 mice per group), as shown in Table 1. Initially, in addition to the normal diet, the mice in the prevention group were given different doses of the polysaccharide solution daily. After 5 d, the mice received 36 h antibiotic streptomycin (5 g/L) treatment, which was added to drinking water containing 6.7% fructose. After 12 h of fasting, 0.2 mL of 0.3% NaHCO_3_ aqueous solution was supplemented to all the mice and 30 min later, the mice were challenged with 10^9^ CFU of ETEC K88 or PBS on the morning of 8–10 d. Infection symptoms, such as weight loss, diarrhea, and the mice feces’ water content, were observed. All mice were sacrificed by cervical dislocation after retro-orbital bleeding was taken 10 d into the experiment. The blood was collected to separate the serum and frozen at −20 °C for testing. The mice’s jejunum tissue was removed in aseptic conditions for scanning electron microscopy (SEM) observation and hematoxylin-eosin (HE) staining.

### 4.5. Diarrhea Indices Determination

The standard for establishing a diarrhea model generally uses diarrhea rate and diarrhea index as the main indicators of diarrhea [65]. The diarrhea rate was calculated as the percentage of diarrhea mice in the total number of mice. The diluted/watery stools were graded as follows: the diameter <1 cm was scored 1 point; 1–1.9 cm was scored 2 points; 2–3 cm was scored 3 points; >3 cm was scored 4 points. For the stools with uniformly round shape, the diameter was measured; for the irregular ones, both the longest and shortest diameters were measured and the average value was calculated. The dilute stool rate of each mouse was calculated as the number of dilute stools divided by the total number of stools. Finally, the diarrhea index was calculated as the dilute stool rate multiplied by the dilute stool level.

### 4.6. Analysis of the Integrity of the Jejunum Villi

The jejunum villi’s integrity was determined using SEM, as described in a previous report [66]. Transverse slices of each intestinal segment (2 mm × 2 mm) were prepared and the contents and intestinal mucus adhered to the surface were washed away by PBS (0.1 mol/L). The jejunum was immersed in 2.5% (*v/v*) glutaraldehyde for fixation overnight. The samples fixed overnight were rinsed with PBS three times for 5 min each. Then, they were immersed in ethanol with a concentration of 30%, 50%, 70%, 80%, 90%, 95%, and 100% for dehydration for 15 min each time. After dehydration, the sample was soaked in a mixture of ethanol–isoamyl acetate (1:1, *v/v*) for 30 min, and then treated with 100% isoamyl acetate for 1 h to replace the ethanol. After being dried, the samples were mounted, coated, and observed by using SEM (Phenom-World PW-100-016, Eindhoven, Netherlands).

### 4.7. Histopathological Observation

The proximal jejunum inflammation in the mice with bacterial diarrhea was investigated via histological assessment, based on a method described in an earlier study [63]. Briefly, on the 10th day, all mice were sacrificed and the proximal jejuna removed. Subsequently, the samples were fixed, embedded, and cut into sections with 5 μm thickness. After being stained with HE, the sections were evaluated by a light microscopy (Olympus BX41, Tokyo, Japan).

### 4.8. Inflammation-Related Factors in the Serum

On the 10th day, vitreous blood was collected to obtain serum for determining the inflammation-related factors. IgA, TNF-α, and mMCP-1 were identified using ELISA kits according to the manufacturer’s instructions.

### 4.9. Detection of Gut Microbiota

The bacterial gut communities’ compositions were examined using 16S rRNA gene sequencing by the Shanghai Majorbio Bio-Pharm Technology Co., Ltd. (Shanghai, China). The fecal samples from the five animal groups were collected and flash-frozen in liquid nitrogen before storage at −80 °C. The extraction and purification of DNA were performed as described previously [67]. The 16S rRNA gene comprising the V3-V4 regions was amplified using a standard primer pair (341F, 805R). Subsequently, a microbial diversity analysis was performed according to a method described in an earlier study [67]. The sequences clustered at 97% similarity were assigned to the same operational taxonomic unit (OTU).

### 4.10. Statistical Analysis

All data were expressed as mean ± SD of at least three individual experiments. Statistical analyses were performed using SPSS Statistical 24 Software. Comparisons between groups were performed using one-way analysis of variance (ANOVA) with Duncan’s range tests. A normality test showed that all the raw data displayed a normal distribution, while a variance test indicated that all groups exhibited equal variance. *p* < 0.05 (two-sided) was regard as significant (*, *p* < 0.05; **, *p* < 0.01; ***, *p* < 0.001).

## 5. Conclusions

This study evaluates the anti-diarrheal activity of DESP on ETEC-K88-induced diarrhea in mice. The results show that DESP can reduce the excretion of diarrheal feces and the accumulation of intestinal fluids, improve weight loss, and reduce the diarrhea rate and diarrhea index in mice. Furthermore, DESP reduces the release of TNF-α and mMCP-1 proinflammatory factors, and the production of serum IgA, repairing intestinal mucosal damage. In addition, at the family level, DESP increases the abundance of *Lactobacillaceae*, *Bifidobacteriaceae*, and *Lachnospiraceae*, and decreases the number of *Enterobacteriaceae* and *Bacteroidaceae*. In conclusion, DESP alters the composition and diversity of the intestinal microbiota in mice with ETEC-K88-induced diarrhea and promotes the reconstruction of the microbial environment, thereby alleviating diarrhea symptoms.

## Figures and Tables

**Table 1:** Treatments for the animal experiment.

Groups	Diet (1–10 d) ^a^	Antibiotic Treatment (6–7 d)	Injection (8–10 d)
PBS	Drinking water		PBS
Model	Drinking water	5 g/L streptomycin in drinking water	10^9^ cfu ETEC
DESP_50_	50mg/kg DESP	5 g/L streptomycin in drinking water	10^9^ cfu ETEC
DESP_100_	100mg/kg DESP	5 g/L streptomycin in drinking water	10^9^ cfu ETEC
DESP_200_	200mg/kg DESP	5 g/L streptomycin in drinking water	10^9^ cfu ETEC

^a^ All mice are freely fed standard breeding feed (Shanghai Slack Laboratory Animal Co., Ltd.) during this period.

## Data Availability

No new data were created or analyzed in this study. Data sharing is not applicable to this article.

## Abbreviations

ETEC	enterotoxigenic *Escherichia coli*
DESP	polysaccharides from *Eucheuma serra*
IECs	epithelial cells
ST	heat-stable enterotoxins
LT	heat-labile toxin
IgA	immunoglobulin A
TNF-α	Tumor necrosis factor-a
MCP-1	Monocyte chemotactic protein 1
OUT	operational taxonomic units
SM	streptomycin
TLR	Toll-like receptors
NF-κB	nuclear factor-κB
ROS	reactive oxygen species
SCFAs	short-chain fatty acid
AAD	antibiotic-associated diarrhea

## References

[1] GBD 2015 Disease and Injury Incidence and Prevalence Collaborators (2016). Global, regional, and national incidence, prevalence, and years lived with disability for 310 diseases and injuries, 1990–2015: A systematic analysis for the Global Burden of Disease Study 2015. Lancet.

[2] Walker R.I. (2015). An assessment of enterotoxigenic *Escherichia coli* and *Shigella* vaccine candidates for infants and children. Vaccine.

[3] Svennerholm A.-M., Tobias J. (2008). Vaccines against enterotoxigenic *Escherichia coli*. Expert Rev. Vaccines.

[4] Nataro J.P., Kaper J.B. (1998). Diarrheagenic *Escherichia coli*. Clin. Microbiol. Rev..

[5] Ruan X., Zhang W. (2013). Oral immunization of a live attenuated *Escherichia coli* strain expressing a holotoxin-structured adhesin–toxoid fusion (1FaeG-FedF-LTA2:5LTB) protected young pigs against enterotoxigenic *E. coli* (ETEC) infection. Vaccine.

[6] Suh J.-S., Hahn W.-H., Cho B.-S. (2010). Recent Advances of Oral Rehydration Therapy (ORT). Electrolyte Blood Press.

[7] Steinway S.N., Biggs M.B., Loughran T.P.J., Papin J.A., Albert R. (2015). Inference of Network Dynamics and Metabolic Interactions in the Gut Microbiome. PLoS Comput. Biol..

[8] Wang S., Wang W., Hou L., Qin L., He M., Li W., Mao W. (2020). A sulfated glucuronorhamnan from the green seaweed *Monostroma nitidum*: Characteristics of its structure and antiviral activity. Carbohydr. Polym..

[9] Cui M., Wu J., Wang S., Shu H., Zhang M., Liu K., Liu K. (2019). Characterization and anti-inflammatory effects of sulfated polysaccharide from the red seaweed *Gelidium pacificum Okamura*. Int. J. Biol. Macromol..

[10] Liang W., Mao X., Peng X., Tang S. (2014). Effects of sulfate group in red seaweed polysaccharides on anticoagulant activity and cytotoxicity. Carbohydr. Polym..

[11] Isnansetyo A., Lutfia F.N.L., Nursid M., Susidarti R.A. (2017). Cytotoxicity of Fucoidan from Three Tropical Brown Algae Against Breast and Colon Cancer Cell Lines. Phcog. J..

[12] Leódido A.C.M., Costa L.E.C., Araújo T.S.L., Costa D.S., Sousa N.A., Souza L.K.M., Sousa F.B.M., Filho M.D.S., Vasconcelos D.F.P., Silva F.R.P. (2017). Anti-diarrhoeal therapeutic potential and safety assessment of sulphated polysaccharide fraction from *Gracilaria intermedia* seaweed in mice. Int. J. Biol. Macromol..

[13] Sousa N.A., Barros F.C., Araujo T.S., Costa D.S., Souza L.K., Sousa F.B., Leodido A.C., Pacifico D.M., de Araujo S., Bezerra F.F. (2016). The efficacy of a sulphated polysaccharide fraction from *Hypnea musciformis* against diarrhea in rodents. Int. J. Biol. Macromol..

[14] Liu B., Liu Q.-M., Li G.-L., Sun L.-C., Gao Y.-Y., Zhang Y.-F., Liu H., Cao M.-J., Liu G.-M. (2019). The anti-diarrhea activity of red algae-originated sulphated polysaccharides on ETEC-K88 infected mice. RSC Adv..

[15] Bezerra F.F., Lima G.C., de Sousa N.A., de Sousa W.M., Costa L.E.C., da Costa D.S., Barros F.C.N., Medeiros J.V.R., Freitas A.L.P. (2018). Antidiarrheal activity of a novel sulfated polysaccharide from the red seaweed *Gracilaria cervicornis*. J. Ethnopharmacol..

[16] Rouhani S., Griffin N.W., Yori P.P., Gehrig J.L., Olortegui M.P., Salas M.S., Trigoso D.R., Moulton L.H., Houpt E.R., Barratt M.J. (2020). Diarrhea as a Potential Cause and Consequence of Reduced Gut Microbial Diversity Among Undernourished Children in Peru. Clin. Infect. Dis..

[17] Dinleyici E.C., Martinez-Martinez D., Kara A., Karbuz A., Dalgic N., Metin O., Yazar A.S., Guven S., Kurugol Z., Turel O. (2018). Time Series Analysis of the Microbiota of Children Suffering from Acute Infectious Diarrhea and Their Recovery After Treatment. Front. Microbiol..

[18] Ma T., Villot C., Renaud D., Skidmore A., Chevaux E., Steele M., Guan L.L. (2020). Linking perturbations to temporal changes in diversity, stability, and compositions of neonatal calf gut microbiota: Prediction of diarrhea. ISME J..

[19] Lv W., Liu C., Ye C., Sun J., Tan X., Zhang C., Qu Q., Shi D., Guo S. (2017). Structural modulation of gut microbiota during alleviation of antibiotic-associated diarrhea with herbal formula. Int. J. Biol. Macromol..

[20] Porse H., Rudolph B. (2017). The seaweed hydrocolloid industry: 2016 updates, requirements, and outlook. J. Appl. Phycol..

[21] Fan Y., Wang W., Song W., Chen H., Teng A., Liu A. (2012). Partial characterization and anti-tumor activity of an acidic polysaccharide from *Gracilaria lemaneiformis*. Carbohydr. Polym..

[22] Liu Y., Liu W., Wang Y., Ma Y., Huang L., Zou C., Li D., Cao M.-J., Liu G.-M. (2019). Inhibitory Effect of Depolymerized Sulfated Galactans from Marine Red Algae on the Growth and Adhesion of Diarrheagenic *Escherichia coli*. Mar. Drugs.

[23] Qiu J. (2007). Traditional medicine: A culture in the balance. Nature.

[24] Xie J.-H., Jin M.-L., Morris G.A., Zha X.-Q., Chen H.-Q., Yi Y., Li J.-E., Wang Z.-J., Gao J., Nie S.-P. (2016). Advances on Bioactive Polysaccharides from Medicinal Plants. Crit. Rev. Food Sci. Nutr..

[25] Dong N., Li X., Xue C., Wang C., Xu X., Bi C., Shan A., Li D. (2019). Astragalus polysaccharides attenuated inflammation and balanced the gut microflora in mice challenged with *Salmonella typhimurium*. Int. Immunopharmacol..

[26] Liu L., Guo Z., Lv Z., Sun Y., Cao W., Zhang R., Liu Z., Li C., Cao S., Mei Q. (2008). The beneficial effect of *Rheum tanguticum* polysaccharide on protecting against diarrhea, colonic inflammation and ulceration in rats with TNBS-induced colitis: The role of macrophage mannose receptor in inflammation and immune response. Int. Immunopharmacol..

[27] Fairbrother J., Nadeau E., Gyles C. (2005). *Escherichia coli* in post-weaning diarrhea in pigs: An update on bacterial types, pathogenesis, and prevention strategies. Anim. Health Res. Rev..

[28] Fleckenstein J.M., Hardwidge P.R., Munson G.P., Rasko D.A., Sommerfelt H., Steinsland H. (2010). Molecular mechanisms of enterotoxigenic *Escherichia coli* infection. Microbes Infect.

[29] Shimazu R., Akashi S., Ogata H., Nagai Y., Fukudome K., Miyake K., Kimoto M. (1999). MD-2, a Molecule that Confers Lipopolysaccharide Responsiveness on Toll-like Receptor 4. J. Exp. Med..

[30] Yang K., Jiang Z., Zheng C., Wang L., Yang X. (2014). Effect of *Lactobacillus plantarum* on diarrhea and intestinal barrier function of young piglets challenged with enterotoxigenic *Escherichia coli* K88. J. Anim. Sci..

[31] Wilmore J.R., Gaudette B.T., Gomez Atria D., Hashemi T., Jones D.D., Gardner C.A., Cole S.D., Misic A.M., Beiting D.P., Allman D. (2018). Commensal Microbes Induce Serum IgA Responses that Protect against Polymicrobial Sepsis. Cell Host Microbe.

[32] Trygstad C., Stiehm E. (1971). Elevated serum IgA globulin in anaphylactoid purpura. Pediatrics.

[33] Whitworth J.A., Leibowitz S., Kennedy M.C., Cameron J.S., Chantler C. (1976). IgA and glomerular disease. Clin. Nephrol..

[34] Ma T.Y., Boivin M.A., Ye D., Pedram A., Said H.M. (2005). Mechanism of TNF-α modulation of Caco-2 intestinal epithelial tight junction barrier: Role of myosin light-chain kinase protein expression. Am. J. Physiol. Gastr. L..

[35] Lawrence C.E., Paterson Y.Y., Wright S.H., Knight P.A., Miller H.R. (2004). Mouse mast cell protease-1 is required for the enteropathy induced by gastrointestinal helminth infection in the mouse. Gastroenterology.

[36] Sun Y., Liu Z., Song S., Zhu B., Zhao L., Jiang J., Liu N., Wang J., Chen X. (2020). Anti-inflammatory activity and structural identification of a sulfated polysaccharide CLGP4 from *Caulerpa lentillifera*. Int. J. Biol. Macromol..

[37] Ji J., Hu S., Zheng M., Du W., Shang Q., Li W. (2013). Bacillus amyloliquefaciens SC06 inhibits ETEC-induced pro-inflammatory responses by suppression of MAPK signaling pathways in IPEC-1 cells and diarrhea in weaned piglets. Livest. Sci..

[38] Valdes A.M., Walter J., Segal E., Spector T.D. (2018). Role of the gut microbiota in nutrition and health. BMJ.

[39] Richards J.L., Yap Y.A., McLeod K.H., Mackay C.R., Marino E. (2016). Dietary metabolites and the gut microbiota: An alternative approach to control inflammatory and autoimmune diseases. Clin. Trans. Immunol..

[40] Liu Y., Ma Y., Chen Z., Li D., Liu W., Huang L., Zou C., Cao M.-J., Liu G.-M., Wang Y. (2020). Antibacterial Activity of Sulfated Galactans from *Eucheuma serra* and *Gracilari verrucosa* against Diarrheagenic *Escherichia coli* via the Disruption of the Cell Membrane Structure. Mar. Drugs.

[41] Guo Q., Goldenberg J.Z., Humphrey C., El Dib R., Johnston B.C. (2019). Probiotics for the prevention of pediatric antibiotic-associated diarrhea. Cochrane Database Syst. Rev..

[42] Barbut F., Meynard J.L. (2002). Managing antibiotic associated diarrhoea. BMJ.

[43] Schatz A., Bugle E., Waksman S.A. (2005). Streptomycin, a substance exhibiting antibiotic activity against gram-positive and gram-negative bacteria. 1944. Clin. Orthop. Relat. Res..

[44] Zhang N., Liang T., Jin Q., Shen C., Zhang Y., Jing P. (2019). Chinese yam (*Dioscorea opposita* Thunb.) alleviates antibiotic-associated diarrhea, modifies intestinal microbiota, and increases the level of short-chain fatty acids in mice. Food Res. Int..

[45] Li S., Qi Y., Chen L., Qu D., Li Z., Gao K., Chen J., Sun Y. (2018). Effects of *Panax ginseng* polysaccharides on the gut microbiota in mice with antibiotic-associated diarrhea. Int. J. Biol. Macromol..

[46] Li S., Qi Y., Ren D., Qu D., Sun Y. (2019). The Structure Features and Improving Effects of Polysaccharide from *Astragalus membranaceus* on Antibiotic-Associated Diarrhea. Antibiotics.

[47] Sánchez B., Delgado S., Blanco-Miguez A., Lourenco A., Gueimonde M., Margolles A. (2017). Probiotics, gut microbiota, and their influence on host health and disease. Mol. Nutr. Food Res..

[48] Hegarty J.W., Guinane C.M., Ross R.P., Hill C., Cotter P.D. (2016). Bacteriocin production: A relatively unharnessed probiotic trait?. F1000Research.

[49] Sanders M.E., Merenstein D.J., Reid G., Gibson G.R., Rastall R.A. (2019). Probiotics and prebiotics in intestinal health and disease: From biology to the clinic. Nat. Rev. Gastroenterol. Hepatol..

[50] Parada Venegas D., De la Fuente M.K., Landskron G., Gonzalez M.J., Quera R., Dijkstra G., Harmsen H.J.M., Faber K.N., Hermoso M.A. (2019). Short Chain Fatty Acids (SCFAs)-Mediated Gut Epithelial and Immune Regulation and Its Relevance for Inflammatory Bowel Diseases. Front. Immunol..

[51] Louis P., Flint H.J. (2017). Formation of propionate and butyrate by the human colonic microbiota. Environ. Microbiol..

[52] Fachi J.L., de Souza Felipe J., Pral L.P., da Silva B.K., Corrêa R.O., de Andrade M.C.P., da Fonseca D.M., Basso P.J., Câmara N.O.S., e Souza É.L.D.S. (2019). Butyrate Protects Mice from *Clostridium difficile*—Induced Colitis through an HIF-1-Dependent Mechanism. Cell Rep..

[53] Furusawa Y., Obata Y., Fukuda S., Endo T.A., Nakato G., Takahashi D., Nakanishi Y., Uetake C., Kato K., Kato T. (2013). Commensal microbe-derived butyrate induces the differentiation of colonic regulatory T cells. Nature.

[54] Donaldson G., Lee S., Mazmanian S. (2015). Gut biogeography of the bacterial microbiota. Nat. Rev. Microbiol..

[55] Hooda S., Boler B.M.V., Serao M.C.R., Brulc J.M., Staeger M.A., Boileau T.W., Dowd S.E., Fahey G.C.J., Swanson K.S. (2012). 454 pyrosequencing reveals a shift in fecal microbiota of healthy adult men consuming polydextrose or soluble corn fiber. J. Nutr..

[56] Sassone-Corsi M., Nuccio S.P., Liu H., Hernandez D., Vu C.T., Takahashi A.A., Edwards R.A., Raffatellu M. (2016). Microcins mediate competition among Enterobacteriaceae in the inflamed gut. Nature.

[57] Wang H., Li Y., Feng X., Li Y., Wang W., Qiu C., Xu J., Yang Z., Li Z., Zhou Q. (2016). Dysfunctional gut microbiota and relative co-abundance network in infantile eczema. Gut Pathog..

[58] Shang Q., Shan X., Cai C., Hao J., Li G., Yu G. (2016). Dietary fucoidan modulates the gut microbiota in mice by increasing the abundance of *Lactobacillus* and *Ruminococcaceae*. Food Funct..

[59] Lagkouvardos I., Lesker T.R., Hitch T.C.A., Gálvez E.J.C., Smit N., Neuhaus K., Wang J., Baines J.F., Abt B., Stecher B. (2019). Sequence and cultivation study of *Muribaculaceae* reveals novel species, host preference, and functional potential of this yet undescribed family. Microbiome.

[60] Satoh-Takayama N., Kato T., Motomura Y., Kageyama T., Taguchi-Atarashi N., Kinoshita-Daitoku R., Kuroda E., Di Santo J.P., Mimuro H., Moro K. (2020). Bacteria-Induced Group 2 Innate Lymphoid Cells in the Stomach Provide Immune Protection through Induction of IgA. Immunity.

[61] Cui M., Zhou R., Wang Y., Zhang M., Liu K., Ma C.-C. (2020). Beneficial effects of sulfated polysaccharide from the red seaweed *Gelidium pacificum* Okamura on mice with antibiotic-associated diarrhea. Food Funct..

[62] Verhelst R., Schroyen M., Buys N., Niewold T. (2014). Dietary polyphenols reduce diarrhea in enterotoxigenic *Escherichia coli* (ETEC) infected post-weaning piglets. Livest. Sci..

[63] Tang C., Xie B., Zong Q., Sun Z. (2019). Proanthocyanidins and probiotics combination supplementation ameliorated intestinal injury in Enterotoxigenic *Escherichia coli* infected diarrhea mice. J. Funct. Foods.

[64] Liu M., Liu Y., Cao M.-J., Liu G.-M., Chen Q., Sun L., Chen H. (2017). Antibacterial activity and mechanisms of depolymerized fucoidans isolated from *Laminaria japonica*. Carbohydr. Polym..

[65] Chen W., Peng X., Yu J., Chen X., Yuan M., Xiang R., He L., Yu D., Kang H., Pan Y. (2020). FengLiao affects gut microbiota and the expression levels of Na+/H+ exchangers, aquaporins and acute phase proteins in mice with castor oil-induced diarrhea. PLoS ONE.

[66] Azumi R., Morita K., Mizutani Y., Hayatsu M., Terai S., Ushiki T. (2018). Dynamics of basal lamina fenestrations in the rat intestinal villous epithelium in response to dietary conditions. Bioend. Res. Tokyo.

[67] Xue M., Ji X., Liang H., Liu Y., Wang B., Sun L., Li W. (2018). The effect of fucoidan on intestinal flora and intestinal barrier function in rats with breast cancer. Food Funct..

